# Dendritic Cell Maturation Regulates TSPAN7 Function in HIV-1 Transfer to CD4^+^ T Lymphocytes

**DOI:** 10.3389/fcimb.2020.00070

**Published:** 2020-02-28

**Authors:** Brieuc P. Perot, Victor García-Paredes, Marine Luka, Mickaël M. Ménager

**Affiliations:** ^1^Inflammatory Responses and Transcriptomic Networks in Diseases, Institut Imagine, Paris, France; ^2^Inserm U1163, Paris, France

**Keywords:** HIV-1, trans-infection, TSPAN7, actin nucleation, dendritic cell maturation, kinetic of transfer

## Abstract

Dendritic cells (DCs) serve a key function in host defense, linking innate detection of microbes to activation of pathogen-specific adaptive immune responses. DCs express cell surface receptors for HIV-1 entry, but are relatively resistant to productive viral replication. They do, however, facilitate infection of co-cultured T-helper cells through a process referred to as trans-infection. We previously showed that tetraspanin 7 (TSPAN7), a transmembrane protein, is involved, through positive regulation of actin nucleation, in the transfer of HIV-1 from the dendrites of immature monocyte-derived DCs (iMDDCs) to activated CD4^+^ T lymphocytes. Various molecular mechanisms have been described regarding HIV-1 trans-infection and seem to depend on DC maturation status. We sought to investigate the crosstalk between DC maturation status, TSPAN7 expression and trans-infection. We followed trans-infection through co-culture of iMDDCs with CD4^+^ T lymphocytes, in the presence of CXCR4-tropic replicative-competent HIV-1 expressing GFP. T cell infection, DC maturation status and dendrite morphogenesis were assessed through time both by flow cytometry and confocal microscopy. Our previously described TSPAN7/actin nucleation-dependent mechanism of HIV-1 transfer appeared to be mostly observed during the first 20 h of co-culture experiments and to be independent of HIV replication. In the course of co-culture experiments, we observed a progressive maturation of MDDCs, correlated with a decrease in TSPAN7 expression, a drastic loss of dendrites and a change in the shape of DCs. A TSPAN7 and actin nucleation-independent mechanism of trans-infection, relying on HIV-1 replication, was then at play. We discovered that TSPAN7 expression is downregulated in response to different innate immune stimuli driving DC maturation, explaining the requirement for a TSPAN7/actin nucleation-independent mechanism of HIV transfer from mature MDDCs (mMDDCs) to T lymphocytes. As previously described, this mechanism relies on the capture of HIV-1 by the I-type lectin CD169/Siglec-1 on mMDDCs and the formation of a “big invaginated pocket” at the surface of DCs, both events being tightly regulated by DC maturation. Interestingly, in iMDDCs, although CD169/Siglec-1 can capture HIV-1, this capture does not lead to HIV-1 transfer to T lymphocytes.

## Introduction

Dendritic cells (DCs) are key players in the mounting of both innate and adaptive immunity. They are present, in an immature state, in organs and can sequentially: engulf elements from their environment; sense engulfed pathogens, which may trigger inflammatory cytokine secretion and their maturation; migrate to lymph nodes and present antigens to T lymphocytes (Merad et al., 2013). Maturation leads to a decrease in antigen uptake, to an increase in major histocompatibility complex and co-stimulatory protein expression and to the secretion of pro-inflammatory cytokines (Alloatti et al., 2016). Peptide-loaded MHCs (pMHCs), co-stimulatory and inflammatory proteins all participate in the stimulation of T cells harboring a cognate receptor to the pMHC allowing the priming of an efficient adaptive immune response (Jain and Pasare, 2017).

DCs of the genital and anorectal tract mucosa are thought to bind and capture HIV-1 and to participate in translocating HIV-1 to patients' *Milieu Interieur*, thereby participating in HIV-1 infection (Ahmed et al., 2015). Interestingly, DCs have also been shown in *in vitro* and *ex vivo* settings, to increase the infection of CD4^+^ T lymphocytes through cis-infection and trans-infection mechanisms (Wu and KewalRamani, 2006; Bertram et al., 2019a). Cis-infection is the process whereby productively-infected DCs produce virus progeny, which augments the amount of virions available to infect CD4^+^ T lymphocytes (Bracq et al., 2018). Trans-infection (sometimes referred to as trans-enhancement) does not involve DC infection but rather the binding of HIV-1 to molecules on DC surface followed or not by the internalization of virions in non-degradative compartments within cells that may or may not communicate with the extracellular milieu (Kijewski and Gummuluru, 2015; Bracq et al., 2018). This binding and/or internalization of HIV-1, with limited infection of DCs, increases CD4^+^ T cell infection *in vitro* and may involve immunological synapse formation (contact zone between DCs and CD4^+^ T cells) thereby polarizing HIV-1 delivery to CD4^+^ T cells (Bracq et al., 2018).

Human DCs express HIV-1 restriction factors among which is SAM domain and HD domain-containing protein 1 (SAMHD1), the major protein responsible for the relative resistance of DCs to HIV-1 infection (Antonucci et al., 2017). SAMHD1 is a phosphohydrolase that is capable of degrading the cellular pool of deoxynucleoside triphosphates, thereby interfering with reverse transcription (Antonucci et al., 2017). Although DCs are relatively resistant to infection by HIV-1, strong DC subtype susceptibility exists (Martin-Gayo and Yu, 2019).

Cyclic GMP-AMP synthase (cGAS) is a sensor for HIV-1 DNA, product of reverse transcription, that is expressed by DCs. DCs however do not respond to HIV-1 infection owing to the block of reverse transcription resulting from SAMHD1 presence (Landau, 2014). Interestingly, when SAMHD1 is degraded experimentally, DCs are able to sense HIV-1 and to produce type I interferons in response to HIV-1 and lose their trans-infection capacity (Manel et al., 2010). Another mechanism by which HIV-1 limits IFN induction in infected DCs and macrophages, is through Vpr and Vif-mediated limitation of TBK1 (TANK-binding kinase 1) phosphorylation, in turn limiting IFN induction (Harman et al., 2015). By limiting its detection by the innate sensors of DCs and using these cells for transfer of virions to T cells (as discussed above), the virus may evade, at least in part, the first line of defense of the immune system in mucosal tissues and use DCs as early cell reservoirs to traffic from the peripheral tissues to lymph nodes to establish and amplify infection of CD4^+^ T cells using the trans-infection process. This hijacking of DCs by HIV-1 is sometimes referred to as the trojan horse hypothesis (Izquierdo-Useros et al., 2010).

Although strong evidence concerning the *in vivo* occurrence of HIV-1 trans-infection are lacking owing to the absence of optimal animal models and obvious difficulties to perform experiments (e.g., poor reconstitution of human myeloid compartment in humanized mouse models), *ex vivo* experiments revealed that endogenous myeloid DCs can trans-infect CD4^+^ T cells with HIV-1 (Shen et al., 2014; Reyes-Rodriguez et al., 2016; Trifonova et al., 2018). Langerhans cells, vaginal epithelium DCs and other mucosal CD11c^+^ DCs have also been shown to transfer HIV-1 to CD4^+^ T lymphocytes in *ex vivo* experiments (Nasr et al., 2014; Pena-Cruz et al., 2018; Bertram et al., 2019b). Interestingly, subcutaneous injection of murine leukemia virus (MLV) or HIV-1 in mice results in their uptake by macrophages expressing CD169/Siglec-1, through interaction with this protein (Sewald et al., 2015). Importantly, MLV does not infect CD169^+^ macrophages in mice but, when loaded with MLV and after injection, these cells participate in infecting B lymphocytes and the two cell types form immunological synapses (Sewald et al., 2015). These observations support the trojan horse hypothesis and reinforce the need for a better comprehension of the mechanisms of HIV-1 transfer from DCs to T cells.

As immature DCs (iDCs) and mature DCs (mDCs) differ in their phenotype, function and localization, as exposed above, HIV-1 may encounter them in both states (e.g., immature circulating DCs, immature and mature mucosal DCs, and mature DCs in the draining lymph nodes of HIV-1–infected individuals). Researchers have proposed different mechanisms of trans-infection from iDCs vs. mDCs. Indeed, iDCs can mediate trans-infection through the interaction of several proteins belonging to the C-type lectin receptor (CLRs) family [such as DC-SIGN, mannose receptor (MR) (also known as CD206), langerin (CD207), and syndecan-3] with HIV-1 GP120 protein (Kijewski and Gummuluru, 2015). These interactions, although not leading to potent HIV-1 fusion/entry, are involved in trans-infection by presenting virions to CD4^+^ T cells; however, it has been proposed that the engagement of this CLRs also efficiently directs bound virions to endolysosomal compartment for degradation (Kijewski and Gummuluru, 2015). DCIR, which is highly expressed by immature monocyte-derived DCs (iMDDCs) in particular, was shown to bind HIV-1 particle leading to an increase of both cis- and trans-infection potential of DCs (Lambert et al., 2008).

In mDCs, CLRs are downregulated while the I-type lectin CD169/Siglec-1 is highly upregulated and was shown to be involved in HIV-1 uptake and subsequent cis- and trans-infection of CD4^+^ T cell through interaction with a-2,3-linked sialic acid residues on the glycosphingolipid GM3 (host cell-derived glycosphingolipid that is incorporated in HIV-1 virions) (Puryear et al., 2013; Akiyama et al., 2017). The exosome secretion pathway has also been proposed as a mechanism for trans-infection by mature DCs (Kijewski and Gummuluru, 2015).

Aiming at better understanding the mechanisms at play during trans-infection, we previously demonstrated that tetraspanin 7 (TSPAN7), a transmembrane protein, is involved, through positive regulation of actin nucleation, in the transfer of HIV-1 from the dendrites of iMDDCs to activated CD4^+^ T lymphocytes without HIV-1 internalization (Ménager and Littman, 2016). TSPANs form a large family of transmembrane proteins that play a scaffolding role, are organized in TSPAN-enriched microdomains (TEMs) and are involved in several biological processes such as cell migration, adhesion and virus entry in cells (Saiz et al., 2018). Some TSPANs, such as TSPAN6 and CD9, have been shown to interfere with the detection of viruses by cells through direct interaction with proteins involved in sensing of PAMPs (Saiz et al., 2018). Other published functions are HIV-1 entry regulation (e.g., CD63 regulates CXCR4 HIV co-receptor expression on cell surface), reverse transcription (e.g., CD81 regulates SAMHD1 turnover), assembly and trans-infection (Suárez et al., 2018). TSPAN7, in addition to its recently discovered role in control of HIV transfer through regulation of actin nucleation (Ménager and Littman, 2016), has been found to be mutated in some forms of X-linked intellectual disabilities, which may be explained by the role of this protein in neuronal morphogenesis (Bassani et al., 2012; Usardi et al., 2017). Indeed, TSPAN7 regulates dendritic spines and filopodia formation in neurons and promotes axonal branching (Bassani et al., 2012; Usardi et al., 2017). This protein is also believed to play a role in cancer as it promotes migration and proliferation of lung cancer cells and limits myeloma tumor development (Cheong et al., 2015; Wang et al., 2018). Of note, TSPAN7, which is highly expressed in pancreatic beta cells, is a target for autoantibodies in patients with type 1 diabetes although contribution of these antibodies in the development of pathology is still unclear (McLaughlin et al., 2016).

Using a CCR5-tropic HIV-1 strain (able to infect both DCs and CD4^+^ T lymphocytes, unlike CXCR4-tropic strains, which do not infect DCs), it has been shown that transfer of HIV-1 from MDDCs occurs in a replication-independent mechanisms at first (in the first 20 h of co-culture with CD4^+^ T lymphocytes) and is taken over by a replication-dependent phase (cis-infection phase) partially relying on HIV replication in MDDCs (Turville et al., 2004). This two-phase model, was later confirmed in *ex vivo* derived Langerhans cells (Harman et al., 2009; Nasr et al., 2014).

Here we show that the TSPAN7/actin nucleation dependent transfer of HIV-1 from the dendrites of iMDDCs, is mostly taking place within the first 20 h of co-culture between CD4^+^ T cells, MDDCs and a CXCR4-tropic replicative-competent HIV-1, expressing GFP in the open reading frame of Nef (X4–HIV-1–GFP), independently of HIV-1 replication. Of note, a CXCR4-tropic virus strain was used to focus on trans-infection occurrence rather than cis-infection as DCs are particularly resistant to infection by these strains (Wu and KewalRamani, 2006). In the course of co-culture experiments, a progressive maturation of MDDCs was observed, leading to a decrease in TSPAN7 expression and to a subsequent loss of dendrites and changes in DC shape. At this stage, a TSPAN7 and actin nucleation-independent mechanism, relying on HIV-1 replication, is at play. We also investigated the contribution of TSPAN7/actin nucleation in HIV-1 transfer from LPS-matured MDDCs and found that, as previously described, this mechanism relies on the capture of HIV-1 by the I-type lectin CD169/Siglec-1 and the formation of big invaginated pockets at the surface of DCs, both events tightly regulated by DC maturation. Interestingly, in iMDDCs, although HIV-1 can be captured by CD169/Siglec-1, this capture does not lead to HIV-1 transfer to T lymphocytes.

## Results

### Tetraspanin 7 and Actin Nucleation Are Involved in Early Replication-Independent Steps of HIV-1 Transfer From Immature MDDCs to Activated CD4^+^ T Lymphocytes

**Figure 1:**
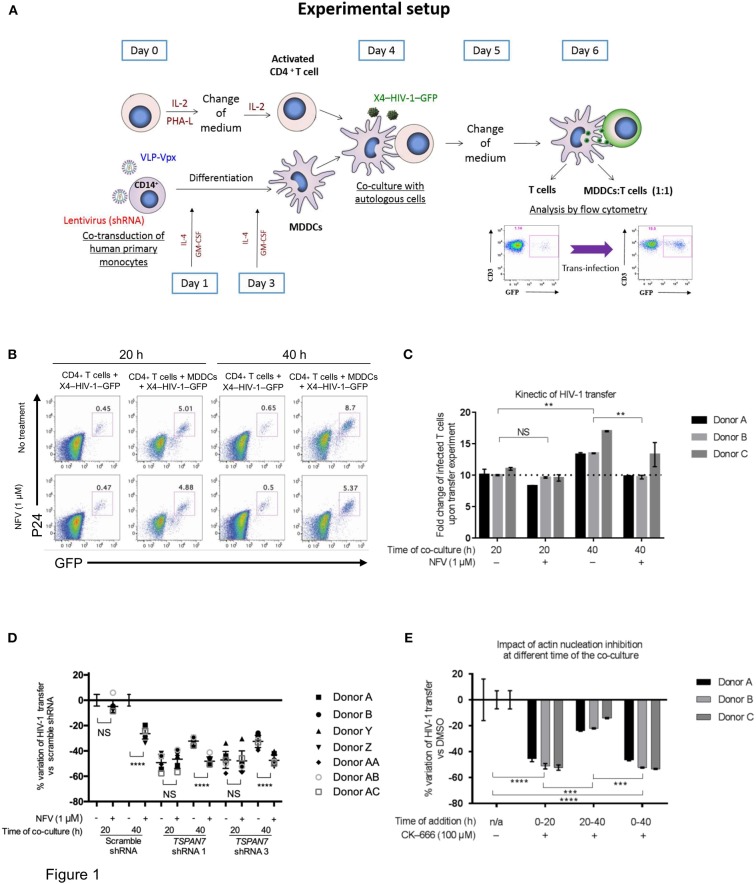
Kinetics of TSPAN7/actin nucleation-dependent mechanism of HIV-1 transfer from immature MDDCs to CD4^+^ T lymphocytes. **(A)** Scheme depicting the experimental layout used throughout this manuscript to assess MDDC-mediated HIV-1 trans-infection of CD4^+^ T cells. MDDC, monocyte-derived dendritic cell; VLP, virus-like particles; IL-2, interleukin-2; PHA-L, phytohaemagglutinin/leucoagglutinin; IL-4, interleukin-4; GM-CSF, granulocytes-macrophage colony-stimulating factor. **(B)** Flow cytometry plots showing GFP and P24 expression levels in cells pre-gated on SSC FSC, living CD3^+^ singlets, 20 or 40 h after the start of co-culture; percentage of CD4^+^ T cells infected by X4–HIV-1–GFP (GFP^+^ and P24^+^) is shown above the gates. Top panel: T cells were cultured with X4–HIV-1–GFP for 20 or 40 h in the absence or presence of MDDCs at a 1:1 ratio and with (bottom panel) or without (top panel) 1 μM Nelfinavir (NFV; an HIV protease inhibitor). **(C)** Bar graph showing the fold increase in T cell infection by X4–HIV-1–GFP following co-culture with MDDCs as compared to CD4^+^ T cells alone [i.e., % infected T cells (GFP^+^ P24^+^) cultured with MDDCs/% infected T cells without MDDCs] as measured by flow cytometry (see **B**). For example, the first bar graph on the left means that, for donor A, 10 times more CD4^+^ T cells were infected when MDDCs were present than when T cells were cultured alone with X4–HIV-1–GFP. Mean ± Standard Deviation (SD) of technical triplicates are presented for three healthy blood donors **(D)** Percentage of variation of X4–HIV-1–GFP transfer with MDDCs transduced with two different shRNAs against *TSPAN7* (shRNA 1 and 3) previously validated (Ménager and Littman, 2016) vs. a non-specific shRNA (scramble shRNA), observed by flow cytometry at 20 or 40 h of co-culture. Dot plots on the right of each time point represent variation of transfer when, in addition to knockdown, 1 μM of NFV was added to the co-culture. Mean ± Standard Deviation (SD) of seven healthy blood donors in the context of 4 independent experiments. **(E)** Bar graph showing the percent variation of X4–HIV-1–GFP transfer (based on fold increase in CD4^+^ T cell infection) between condition where DMSO (drug carrier) was used [0% variation ± Standard Deviation (SD)] vs. 100 μM CK-666 to inhibit the ARP2/3 complex and actin nucleation. CK-666 was added during the first or the last 20 h of co-culture or during the entire experiment. The experiment was performed in three unrelated healthy blood donors, with the mean ± SD of technical triplicates in each condition and for each donor. This experiment is representative of three independent experiments. **(C–E)** NS, not significant. ^**^p < 0.01; ^***^p < 0.001; ^****^p < 0.0001.

To monitor HIV-1 transfer from iMDDCs to CD4^+^ T lymphocytes and the contribution of TSPAN7 relative to the kinetic of transfer and HIV-1 replication, we used an experimental model of co-culture between MDDCs differentiated from circulating monocytes of healthy donors, autologous CD4^+^ T lymphocytes activated by PHA and Il-2 (at a 1:1 ratio) with a replicative-competent CXCR4-tropic HIV-1 encoding the GFP in place of Nef (X4–HIV-1–GFP) (Ménager and Littman, 2016) in the presence or absence of an HIV protease inhibitor (Nelfinavir, NFV), to block HIV replication (Figure 1A, Figure S1A). We observed that most of the T lymphocytes (around 75% of the total number of GFP-expressing T cells, measured after 40 h) were becoming infected during the first 20 h of co-culture, independently of NFV treatment (Figures 1B,C, Figure S1B). A second phase of HIV-1 transfer, which was dependent on HIV-1 replication (blocked by NFV) was identified during the last 20 h of the co-culture (Figures 1B,C, Figure S1B). As we previously demonstrated (Ménager and Littman, 2016), TSPAN7 impacts HIV-1 transfer from iMDDCs to CD4^+^ T cells and most of its effect was observed during the first 20 h, independently of HIV-1 replication (Figure 1D, Figures S1C–E). The Actin Related Protein 2/3 (ARP2/3) inhibitor CK-666 (which can efficiently reduce HIV-1 transfer by blocking actin nucleation; Ménager and Littman, 2016) has the same kinetic of action on X4–HIV-1–GFP transfer as TSPAN7, with most of its effect observed during the first 20 h of co-culture (Ménager and Littman, 2016; Figure 1E, Figure S1F).

Taken together, these results suggest that the previously reported role of TSPAN7 on HIV-1 transfer, through positive regulation of actin nucleation, is mostly effective during the first 20 h of co-culture, in a replication-independent phase, during which most of the T cells are getting infected. TSPAN7 has limited, if any effect on a second phase (from 20 to 40 h of co-culture) of T cells infection, which relies on HIV-1 replication.

### A Change in Shape and Number of Dendrites Is Observed Among MDDCs During Co-culture Experiments

**Figure 2:**
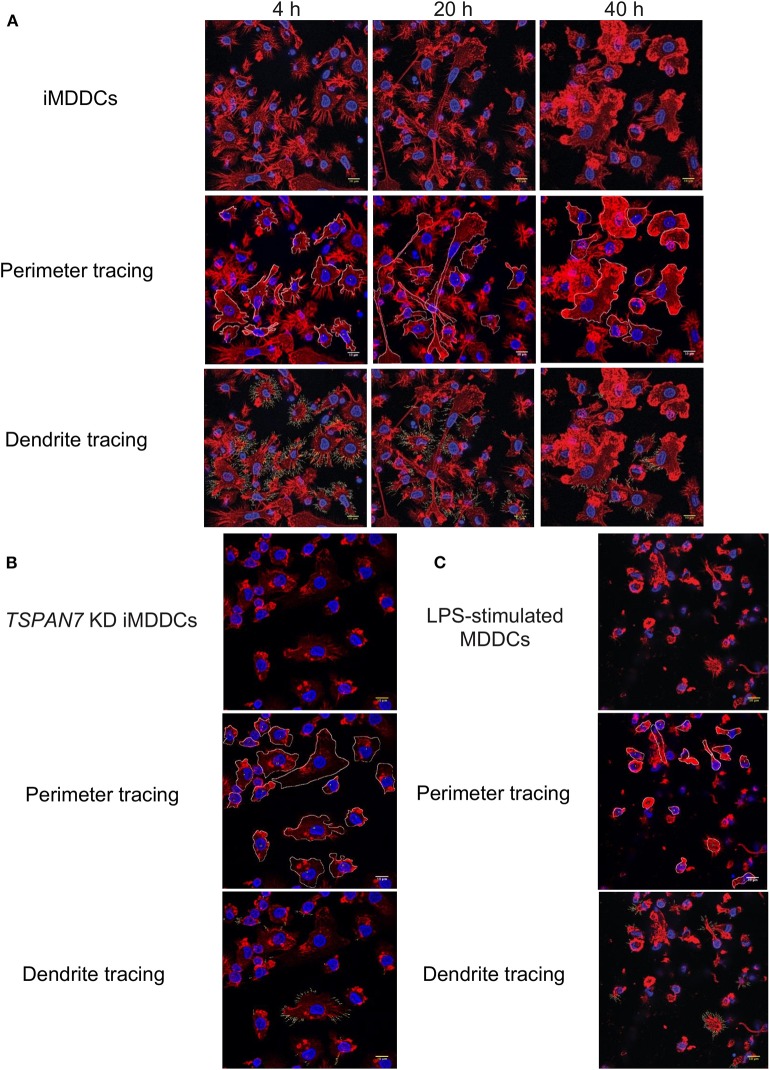
Change of shape and loss of dendrites for MDDCs during HIV-1 transfer experiments. **(A)** Four hundred nanometers of Z-stacks showing by confocal microscopy, iMDDCs and CD4^+^T cells co-cultured with X4–HIV-1–iGag-GFP for 4, 20, and 40 h. Actin was detected in red following phalloidin staining and nuclei stained in blue by DAPI. Using ImageJ software, perimeter of each MDDC was manually traced in white (middle panel) alongside dendrites (bottom panel). **(B)** Same as in **(A)** with iMDDCs transduced with *TSPAN7* shRNA. **(C)** Same as in **(A)** with MDDCs stimulated with 1 μg/ml LPS for 24 h before analysis by confocal microscopy. **(A–C)** Data are from a donor representative of five unrelated donors studied in three independent experiments.

As the role of TSPAN7 in HIV-1 transfer was reported to be through actin-rich dendrites formation (Ménager and Littman, 2016), we next sought to monitor MDDCs morphology and dendrite formation at 4, 20, and 40 h following initiation of HIV-1 trans-infection experiments. Through actin staining, we observed a drastic change in MDDC morphology between the first and second 20 h of co-culture (Figure 2A). An increase of MDDC size in general and of their cytosol in particular, correlated with a drastic loss of dendrites, could be seen during the last 20 h of co-culture (Figure 2A). Of note, the morphology of MDDCs at the end of the transfer experiments resembles the one observed in MDDCs following *TSPAN7* knockdown by shRNA (Figure 2B). Stimulation of MDDCs with lipopolysaccharide (LPS) a TLR4 agonist, also resulted in a profound cell morphology and shape modification (Figure 2C). In order to quantify these changes in morphology, we used a circularity index based on changes of perimeter and area (Figure S2A) and quantified the length and number of dendrites observed by confocal microscopy. As a result, between 20 and 40 h, an increase of MDDCs circularity index could be quantified and was reaching levels observed following decrease of *TSPAN7* expression through shRNA or after LPS treatment (Figure S2B). This change in shape observed during the last 20 h was accompanied by a drastic reduction of size and number of dendrites per cell (Figures S2C–E).

### Tetraspanin 7 Expression Is Downregulated During Dendritic Cell Maturation

**Figure 3:**
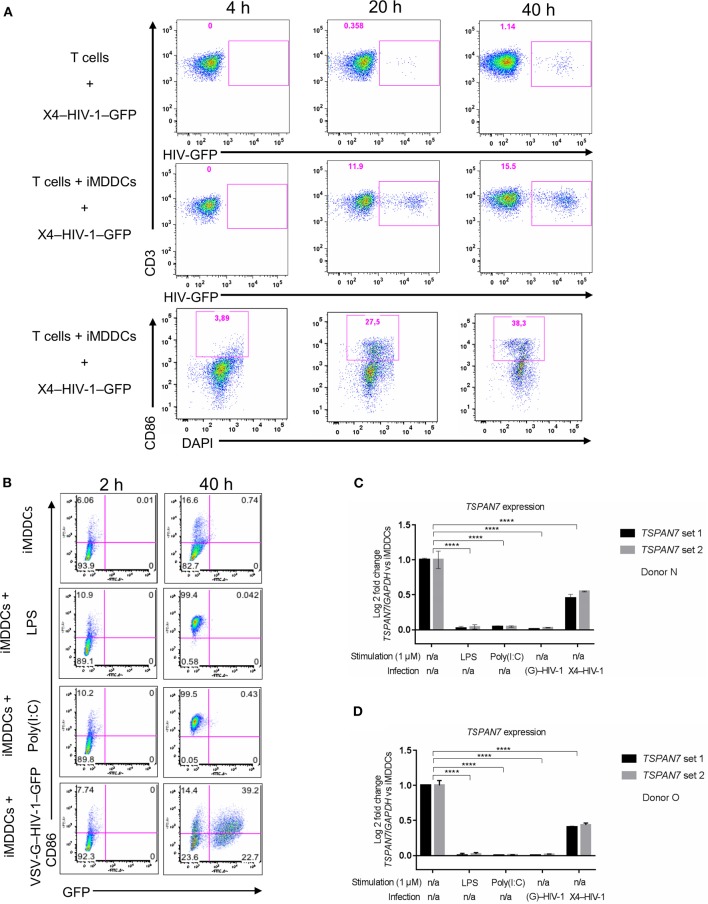
Impact of MDDC maturation on *TSPAN7* expression and function. **(A)** Flow cytometry plots showing CD4^+^ T cells infected with X4–HIV-1–GFP, in the absence (top panel) or presence (middle panel) of iMDDCs. In the bottom panel, the maturation state of MDDCs was evaluated by gating on CD86 expression, upon co-culture with CD4^+^ T cells and X4–HIV-1–GFP measured at 4, 20, and 40 h. Cells were pre-gated as follows: SSC FSC, singlets, living cells, CD3^+^ T cells (top and middle panel), or DC-SIGN^+^ MDDCs (bottom panel) **(B)** Maturation state of MDDCs at 2 and 40 h following treatment with Poly(I:C) (1 μg/ml), LPS (1 μg/ml) or infection with a VSV-G-pseudotyped single-round HIV-1–GFP (VSV–G-HIV-1-GFP), represented as (G)–HIV-1 on the figure, in the presence of the viral protein Vpx to allow infection and innate sensing. CD86 monitoring by flow cytometry was used to assess maturation status and GFP expression for HIV replication. MDDCs were pre-gated following the same gating strategy as mentioned in **(A)**. **(C,D)** Quantitative PCR (qPCR) measurement of the Log2 fold change of *TSPAN7* mRNA normalized by the housekeeping gene *GAPDH*, 40 h after MDDCs stimulation by LPS (1 μg/ml)/Poly(I:C) (1 μg/ml), infection with VSV-G–HIV-1–GFP + Vpx or in the presence of X4–HIV-1–GFP (represented as X4–HIV-1) or left unstimulated. Fold change expression was normalized to the level of *TSPAN7* detected in iMDDCs. Experiments were performed 3 times in 6 independent blood donors. Donor N in **(C)** and donor O in **(D)** are representative of an experiment performed in six unrelated blood donors in the context of six independent experiments, using two different sets of qPCR primers to detect specific expression of *TSPAN7*. NS, not significant. ^****^p < 0.0001.

After studying the kinetic of HIV-1 transfer and changes in cell morphology, we observed, by flow cytometry, an increase in CD86 expression at the surface of MDDCs (an indicator of DC maturation), which was particularly pronounced during the last 20 h of the co-culture experiments (Figure 3A, Figures S3A,B). We next sought to investigate the impact of MDDC maturation status on *TSPAN7* expression, following various innate immune stimuli, such as LPS, polyinosinic:polycytidylic acid [poly(I:C)], a synthetic double-stranded RNA-mimicking (TLR3 agonist). We also induced MDDCs maturation through productive infection of iMDDCs with vesicular stomatitis virus-pseudotyped HIV-1 expressing GFP (VSV-G–HIV-1–GFP), delivered together with virus-like particle carrying the HIV-2 protein Vpx, which allows SAMHD1 degradation thereby facilitating HIV RNA reverse transcription, innate sensing by cGAS, type I interferon production and subsequent MDDC maturation (Manel et al., 2010; Figure 3B). As previously reported (Ménager and Littman, 2016), we detected an increase in the expression of *TSPAN7* during the differentiation of monocytes into iMDDCs, which was drastically shutdown upon MDDC maturation through various innate stimuli (Figures 3C,D). This shutdown of *TSPAN7* expression was also observed after co-culture between iMDDCs and the X4–HIV-1–GFP used during transfer experiments (Figures 3C,D). A specific downregulation of *TSPAN7* expression, as compared to its paralog *TSPAN6* was confirmed by bulk RNA sequencing following DC maturation, in response to the same innate stimuli [LPS, poly(I:C) and VSV-G–HIV-1–GFP; Figure S3C].

These results suggest that the changes in cell morphology and in the number of dendrites observed during the last 20 h of HIV-1 transfer experiments, are due to a decrease in actin nucleation activity, driven by the decrease of TSPAN7 expression, observed subsequently to MDDCs maturation (Figure S3D).

### Tetraspanin 7 and Actin Nucleation Have Limited Impact on Trans-infection of T Lymphocytes by Mature MDDCs

**Figure 4:**
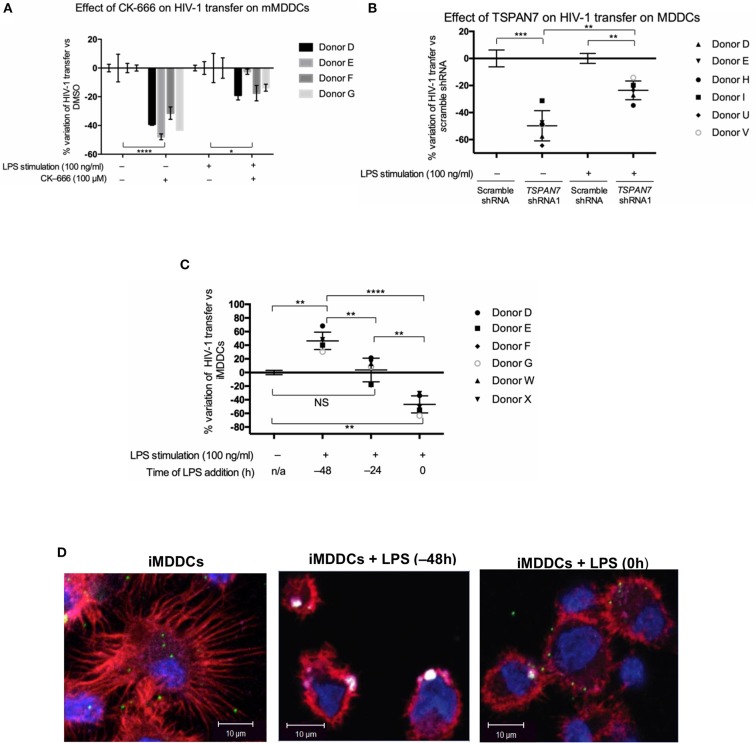
Role of TSPAN7 and actin nucleation in HIV transfer from mature MDDCs to T cells. **(A)** Percentage change of HIV-1 transfer following inhibition of actin nucleation by CK-666 (100 μM), compared to DMSO-treated cells; in immature MDDCs (iMDDCs) and MDDCs matured by 100 ng/ml LPS, 48 h before transfer experiment (mMDDCs). Mean ± SD of triplicates for four different healthy blood donors are represented. **(B)** Percentage change of X4–HIV-1–GFP transfer following *TSPAN7* knockdown compared to scramble shRNA-expressing cells, in iMDDCs and MDDCs matured by 100 ng/ml LPS, 48 h before transfer experiments. Mean ± SD of six different healthy blood donors. **(C)** Percentage change of X4–HIV-1–GFP transfer according to kinetic of maturation of MDDCs [LPS added 48 or 24 h before co-culture or at the time of co-culture (time 0)], compared to iMDDCs. Mean ± SD of six different healthy blood donors. **(A–C)** NS, not significant. ^*^p < 0.05; ^**^p < 0.01; ^***^p < 0.001; ^****^p < 0.0001. **(D)** Confocal microscopy images of MDDCs at different stages of maturation: iMDDCs (left panel); mMDDCs stimulated by LPS (100 ng/ml) 48 h before (middle panel) or at the same time of start of co-culture (right panel). Actin filaments and nuclei were stained with phalloidin (red) and DAPI (blue), respectively and Siglec-1/CD169 was detected using an anti-CD169 antibody conjugated to APC (magenta). Incoming X4–HIV-1–Gag-iGFP particles are seen in green based on (GFP presence). Four hundred nanometers of Z-stacks were taken 20 h after the start of the co-culture with CD4^+^ T cells and X4–HIV-1–Gag-iGFP. The pictures presented here are from a representative donor from four unrelated blood donors.

In the next set of experiments, we interrogated actin nucleation and TSPAN7 function in HIV-1 transfer from LPS-matured MDDCs (mMDDCs) to activated CD4^+^ T lymphocytes. Our results show only a limited contribution of actin nucleation and TSPAN7 when HIV-1 was transferred from mature MDDCs (Figures 4A,B, Figures S4A,B). As previously described (McDonald, 2010), mMDDCs, in our co-culture experiments, were capable of infecting more T lymphocytes through HIV-1 transfer, as compared to iMDDCs, although timing of MDDCs maturation seemed to be key (Figure 4C). Stimulating MDDCs, with LPS 48 h before HIV-1 transfer experiments was required to obtain optimal HIV-1 transfer, and correlated with the appearance of large HIV-1/CD169 (Siglec-1) puncta at the surface of MDDCs, resembling the “big invaginated pocket,” previously reported to be responsible for HIV-1 transfer (Figure 4D, middle panel; McDonald, 2010). Adding LPS at the same time of co-culture experiments, resulted in the loss of dendrites, with a limited formation of Siglec-1/CD169-puncta and an overall decrease in X4–HIV-1–GFP transfer when compared to iMDDCs (Figure 4D, right panel; Figures S4D,E). Although LPS-treated MDDCs were able to capture at least 10 times more viruses than their immature counterparts (Figure S4C), the TSPAN7-independent mechanism of HIV transfer appeared to be 5–10 times less efficient than the one observed in iMDDCs with, at most, an increase of only 50% (1.5-fold) in infected T cells (Figure S4D).

### In Co-culture Experiments, CD169/Siglec-1 Mediates Transfer of HIV-1 From Mature MDDCs but a Different Receptor Is Required for Optimal Transfer From Immature Cells

**Figure 5:**
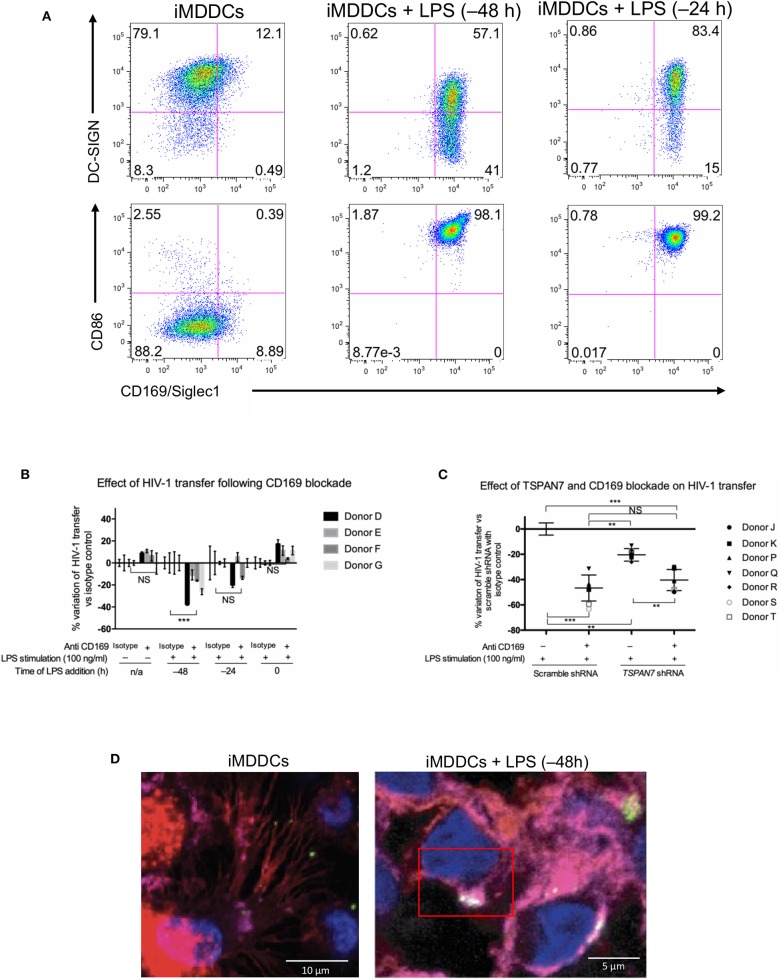
CD169, as an HIV-1 receptor, mostly impacts transfer from mature MDDCs rather than immature MDDCs. **(A)** Flow cytometry plots showing CD86, DC-SIGN, and CD169 expression levels on MDDCs (pre-gated on SSC FSC, living cells, CD3^−^ cells and singlets). Panels show the expression of these proteins in iMDDCs (left panel) and MDDCs with LPS pretreatment at 100 ng/ml for 48 or 24 h before co-culture (middle and right panels, respectively). **(B)** Percentage of variation of HIV-1 transfer when using iMDDCs or LPS-treated MDDCs (100 ng/ml LPS for different lengths of time) incubated with a blocking antibody against CD169 as compared to an isotype control for each condition. Results are displayed for 4 different blood donors with the mean ± SD of technical triplicates. **(C)** Percent of variation in HIV-1 transfer to assess the impact of blocking CD169 and *TSPAN7* knockdown as compared to scramble shRNA on MDDCs matured with LPS for 48 h treated by an isotype control. Mean ± SD of seven different blood donors in 4 experiments. **(B,C)** NS, not significant. ^**^p < 0.01; ^***^p < 0.001. **(D)** Confocal microscopy images of iMDDCs (left panel) and mature MDDCs (mMDDCs) right panel, to assess the degree of colocalization between CD169 (magenta) and incoming X4–HIV-1–Gag-iGFP (green). Actin filaments and nuclei were stained with phalloidin (red) and DAPI (blue). Four hundred nanometers of Z-stacks were taken 40 h after the start of the co-culture with CD4^+^ T cells and X4–HIV-1–Gag-iGFP. The pictures presented here are from a representative donor from four unrelated blood donors.

CD169/Siglec-1 was strongly induced at the surface of MDDCs following LPS stimulation prior to co-culture experiments, whereas a small decrease of DC-SIGN was observed (Figure 5A, Figure S5A). Using anti-Siglec-1/CD169 neutralizing antibodies, we were able to block a significant portion of HIV-1 transfer from fully mature MDDCs (i.e., MDDCs stimulated by LPS 48 h prior to co-culture experiments; Figure 5B, Figures S5B,C). In comparison, although blocking Siglec-1/CD169 at the cell surface with antibodies, led to an almost 4-fold decrease in HIV-1 capture by iMDDCs, it had no impact on HIV-1 transfer (Figure 5B, Figure S5D). Our results suggest that Siglec-1/CD169, although expressed in iMDDCs (at a lower level), cannot efficiently contribute to the transfer of HIV-1 from iMDDCs to CD4^+^ T cells. In comparison, blocking CD169/Siglec-1 in mature MDDCs can have a synergistic effect on the limited impact of knocking down TSPAN7 expression, suggesting, once again, that a Siglec-1/CD169–dependent but TSPAN7-independent mechanism is at play during HIV-1 transfer from mMDDCs to CD4^+^ T lymphocytes (Figure 5C). In line with these results, no colocalization was monitored between CD169 and X4–HIV-1–Gag-iGFP captured at the tip of actin-rich dendrites in immature MDDCs, as compared to the colocalization seen in the big invaginated pocket described in mMDDCs (Figure 5D, Figures S5E–G). Our results support the requirement for another HIV-1 receptor, different from Siglec-1, for the capture of HIV-1, by iMDDCs, at the tips of actin-rich dendrites, followed by subsequent efficient transfer to CD4^+^ T lymphocytes.

## Discussion

**Figure 6:**
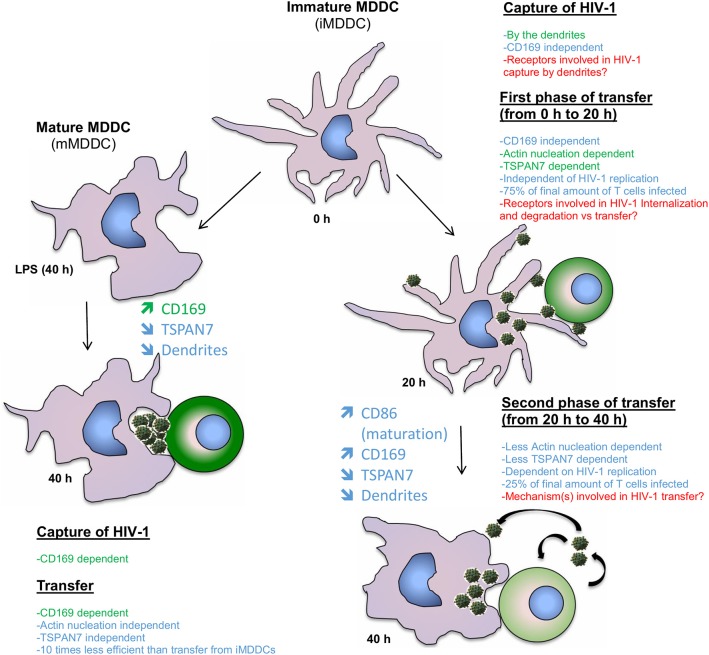
Model comparing the contribution of TSPAN7 and actin nucleation during the different identified phases of HIV transfer when performing co-culture experiments with iMDDCs vs. LPS-matured MDDCs. On the left: mechanism showing the transfer of HIV-1 from LPS-matured MDDCs to CD4^+^ T lymphocytes; as described in the literature, the transfer relies mostly on the capture of HIV-1 by Siglec-1/CD169 and the formation of a big invaginated pocket. TSPAN7 and actin nucleation roles seem rather limited. On the right, a first phase of transfer, dependent on TSPAN7, actin nucleation and dendrite formation is observed during the first 20 h of co-culture with iMDDCs. Seventy-five percent of the transfer is taking place during this first phase, independently of HIV-1 replication and capture by CD169/Siglec-1. A second phase of transfer, less dependent on TSPAN7 and actin nucleation, but dependent on HIV-1 replication is observed during the last 20 h of co-culture. Of note, a decrease of *TSPAN7* expression correlated with a maturation of MDDCs was detected after 20 h of co-culture and leads to a decrease in actin nucleation, inducing a shortening of dendrite and a change in shape of the cells. Previously reported scientific data are shown in green and the new results presented in this manuscript are in blue. Points that will require further investigation in this model are stated in red.

By studying the kinetic of HIV-1 transfer from iMDDCs to CD4^+^ T lymphocytes, we revealed that the previously described role of TSPAN7 and actin nucleation in dendrites formation, required for an efficient transfer, was mostly happening during the first 20 h of co-culture experiments (see proposed model in Figure 6). A second phase of HIV-1 transfer, observed during the next 20 h of co-culture experiments and accounting for less than a quarter of total T cells infection, is happening in an HIV replication-dependent manner and seems to rely less on TSPAN7 and actin nucleation. We observed that this second phase of HIV-1 transfer was accompanied by a drastic change in DC size and a reduction of actin-rich dendrites due to a decrease in TSPAN7 expression subsequent to DC maturation (Figure 6). We confirmed that the transfer of HIV-1 from LPS-matured MDDCs, in co-culture experiments, as previously described in other experimental settings (Landau, 2014), is mostly dependent on the capture of HIV-1 by CD169/Siglec-1 and the formation of a “big invaginated pocket,” rather than through TSPAN7 and actin nucleation. We therefore revealed and confirmed the existence of at least two different mechanisms of HIV-1 transfer in co-culture experiments, depending on MDDCs maturation status: (a) a TSPAN7 and actin nucleation dependent and CD169/Siglec-1–independent mechanism in immature cells vs. (b) a CD169/Siglec-1–dependent, and less TSPAN7 and actin nucleation-dependent process in LPS-matured MDDCs (Figure 6). Of note, although the same level of CD169/Siglec-1 induction at the cell surface following MDDCs maturation with LPS during 24 or 48 h prior to transfer experiments, a strong CD169/Siglec-1-dependent transfer is only measured when maturation is initiated 48 h before co-culture experiments. Based on our observations and quantifications performed by confocal microscopy, we believe that this may reflect an incomplete formation of the big invaginated pocket proposed previously (McDonald, 2010), after only 24 h LPS treatment, rather than a weaker induction of CD169/Siglec-1 expression.

We confirmed that, in our co-culture experiments, as previously demonstrated (McDonald, 2010), mMDDCs can transfer more HIV-1 to activated T lymphocytes than iMDDCs. However, although mMDDCs capture at least 10 times more viral particles, the infection of T cells is only increasing by 1.5-fold, suggesting that the mechanism of transfer used by iMDDCs, through actin-rich dendrites is far more efficient than the CD169/Siglec-1–dependent big invaginated pocket mechanism.

In our experimental settings, to study the transfer of HIV-1 from DCs to T cells, instead of first loading DCs with HIV-1, washing away unbound viruses and co-culturing with activated CD4^+^ T cells, we have chosen, as we previously described, to co-culture at the same time, replicative-competent HIV-1, MDDCs and T cells (Ménager and Littman, 2016). We think that this model can better account for situations potentially encountered at mucosal surfaces where few HIV-1 particles may be captured by iMDDCs actin-rich dendrites and efficiently transferred from the tips of the dendrites to CD4^+^ T cells present in the surrounding areas. Virological synapses would then be established creating an environment where DCs, T cells and HIV-1 are dynamically interacting altogether.

As we did previously, we used DCs derived from human primary monocytes obtained from healthy donors, as an *in vitro* model to study HIV-1 transfer (Ménager and Littman, 2016). Compared to conventional circulating DCs isolated from blood (cDC1, cDC2), mucosal DCs such as Langherans cells and the more recently describe circulating pre-DCs (which may play an important role in HIV-1 physiopathology; Ruffin et al., 2019), MDDCs can be obtained in large quantities and are easier to transduce with lentiviruses. Although it would be really interesting to compare the roles played by the different subsets of isolated blood DCs, they are quite susceptible to experimental manipulation, and studying them in their immature state represents quite a challenge. Recent data indicate that pre-DCs may be one of the key circulating myeloid cell subsets capable of productive infection and trans-infection as they constitutively express CD169/Siglec-1 (Ruffin et al., 2019); as such, they may constitute a promising cell type to investigate when studying trans-infection. Anyhow, in non-inflamed conditions, it is thought that some DC subsets from the dermis and the intestine originate from monocytes, and that, in inflamed tissues, inflammatory DCs are generated from monocytes (Guilliams et al., 2014). MDDCs therefore represent putative DC subtypes that may encounter HIV-1 in the case of infection and in the chronic phase of infection, which leads to persistent inflammation, even under antiretroviral therapy (ART) (Zicari et al., 2019). Of note, *ex vivo* generated MDDCs were shown to be transcriptionally similar to CD14^+^ dermal myeloid cells, which were first thought to be a subtype of DCs, but have been recently demonstrated to be short-lived monocyte-derived macrophages (Harman et al., 2013; McGovern et al., 2014). MDDCs generated *ex vivo* from primary monocytes also represent a well-established model to study both HIV-1 transfer and innate sensing by DCs (Manel et al., 2010).

For this study, we have used, as previously reported (Ménager and Littman, 2016), a CXCR4-tropic HIV-1 strain. Due to the lack of expression of CXCR4 at the surface of DCs, these viruses have even more limited capacity to enter and infect DCs, allowing us to focus on trans- (by monitoring only the transfer of incoming viral particles) rather than cis-infection (due to the transfer of newly production HIV-1 in DCs). We have observed that TSPAN7 and actin nucleation were also involved in DC-mediated trans-infection of CD4^+^ T lymphocytes using transmitted/founder strains of HIV-1 (data not shown), which are CCR5-tropic and infect DCs more efficiently (Kariuki et al., 2017; Bertram et al., 2019a). Interestingly, as mentioned in the introduction, a two-phase trans-infection model has already been proposed for CCR5-tropic HIV-1 strains with the late phase being virus replication-dependent in *in vitro*-generated MDDCs and in mucosal DCs studied *ex vivo* (Turville et al., 2004; Harman et al., 2009; Nasr et al., 2014).

It is interesting to think about the existence of different mechanisms of HIV-1 transfer depending on the maturation status of DCs. DCs are known to switch from being professional pathogens catchers, in their immature state, to potent antigen presenters upon maturation and are then able to establish long-lasting contacts to potently stimulate T cells thereby bridging innate and adaptive immunity (Merad et al., 2013). HIV-1 may have evolved strategies to adapt itself to the changes happening during DC maturation, to exploit DC functions and to be efficiently captured and transferred to T lymphocytes with minimal detection by the immune system. In immature DCs, the actin-rich dendrites would probe the environment and capture HIV-1 through a receptor different from CD169/Siglec-1, which remains to be determined. The virus could then be transferred through dendrites to surrounding T cells, which may result, at some point, as we have observed in our experiments, in DC maturation. DC maturation in turn will lead to some morphological and functional changes, which correlates with an increase in expression of CD169/Siglec-1, a shortening of dendrites and the formation of a big invaginated pocket, which may accumulate HIV-1. Mature DCs, as reported, would then migrate into lymph nodes with a high density of T cells, form long lasting virological synapses with T cells, allowing a CD169/Siglec-1-dependent, TSPAN7/actin nucleation-independent process of HIV-1 transfer to T cells (Kijewski and Gummuluru, 2015; Reyes-Rodriguez et al., 2016). As it has been shown that human DCs are relatively resistant to innate sensing, due to a blockade of HIV-1 viral RNA reverse transcription by SAMHD1, hence of HIV-1 infection, all processes of HIV-1 transfer could happen with minimal stimulation of the innate system, making DCs as the perfect trojan horses for HIV-1 (Antonucci et al., 2017).

As observed in our experiments, during the last 20 h of co-culture, maturation of DCs is happening alongside an increase in circularity and a shortening of the dendrites. These changes are linked to a decrease in actin nucleation activities subsequent to a decrease in TSPAN7 expression. This control of TSPAN7 expression does not seem to be specific to our co-culture experiments, as it is induced by a bacterial TLR4 agonist (LPS), synthetic dsRNA [poly(I:C)] and also observed upon HIV-1 sensing experiments. It would be interesting to unravel the molecular mechanisms by which TSPAN7 expression is shutdown during/as a result of the maturation process, as, to our knowledge, it has not been reported yet in any members of the mammalian tetraspanin family. In particular, we have shown in our study, that TSPAN6, a paralog of TSPAN7, does not seem to be regulated by the same immune stimuli. Such a regulation upon DC maturation also suggests that it may be interesting to investigate TSPAN7 roles in other functions of DCs, in particular the ones modulated upon DC maturation, such as mobility, antigen presentation, production of inflammatory cytokines and expression at the surface of co-stimulatory molecules. We believe that, in our co-culture experiments, the maturation of MDDCs may occur through paracrine or autocrine effects of pro-inflammatory cytokines that may be secreted by T lymphocytes or DCs themselves, initiation of co-stimulatory signals or through microvesicles secreted alongside HIV-1 virions as previously demonstrated (Mercier et al., 2013).

As mentioned in the introduction, most of the experiments demonstrating that trans-infection is a potent way for HIV-1 to propagate have been done in *in vitro* or *ex vivo* settings. However, some experiments performed in *in vivo* conditions suggest that trans-infection may be key for the virus to disseminate *in vivo* especially at early stages of infection and to escape ART agent actions (Bracq et al., 2018). We therefore think that our work contributes to a better understanding of the different mechanisms at play during HIV transfer and highlights the importance of DC maturation status. Such knowledge is required to allow the development of new approaches to tackle HIV-1.

## Materials and Methods

### Cells

HEK293FT cells (Invitrogen) were cultured in DMEM (Gibco), 10% fetal bovine serum (FBS, heat inactivated) (Sigma), 0.1 mM MEM non-essential Amino Acids, 6 mM L-glutamine, 1 mM MEM sodium pyruvate, and penicillin/streptomycin/gentamycin (Gibco).

Peripheral blood mononuclear cells were isolated from buffy coats obtained from healthy human blood donors [Etablissemnt Français du Sang (EFS), agreement N°18/EFS/030] using mononuclear cell isolation with Ficoll-Paque separation media (GE Healthcare). Monocytes were obtained after purification of PBMCs with anti-human CD14 magnetic beads (Miltenyi) and cultured in RPMI (Gibco), 10% FBS (heat inactivated, Sigma), 10 mM Hepes, 55 μM β-mercaptoethanol, 6 mM L-glutamine and penicillin/streptomycin/gentamycin (Gibco) in the presence of 10 ng/mL of human recombinant GM-CSF and 50 ng/mL of human recombinant IL-4 (Miltenyi), in order to differentiate them in monocytes derived dendritic cells (MDDCs). CD4^+^ T lymphocytes were isolated from the CD14^−^ fraction after a purification using anti-human CD4 magnetic beads (Miltenyi). Cells were cultured in the same medium as MDDCs but activated using 2 μg/mL phytohaemagglutinin (PHA-L; eBioscience 00-4977-03) for 48 h combined with human IL-2 treatment (100 U/ml; Miltenyi biotec, 130-097-742). Purity was checked by flow cytometry for both CD14^+^ and CD4^+^ cells and was more than 99%. Fresh media and cytokines were added after 24 and 72 h. By day 4, monocytes were differentiated into MDDCs and were mixed with activated autologous CD4^+^ T lymphocytes and HIV-1 for transfer experiments. MDDC differentiation was assessed by flow cytometry based on DC-SIGN upregulation and CD14 downregulation.

### Plasmids and Viral Particle Production

X4–HIV-1–GFP is a fully replication-competent virus derived from the NL4-3 clone with enhanced Green Fluorescent Protein (GFP) (Clontech, Palo Alto, California, United States) cloned in place of the Nef gene, as previously described (Unutmaz et al., 1999; Motsinger et al., 2002); X4-HIV-Gagi-GFP is derived from the same clone modified to insert the GFP protein between the MA and CA domains of Gag as previously described (Hübner et al., 2007); pLK0.1 GFP was generated by replacing the puromycin resistance gene by GFP in the pLK0.1 puro plasmid (Moffat et al., 2006).

The protein Vpx, as described in Mangeot et al. (2002) and Manel et al. (2010) was delivered using pSIV3+ to generate VSV-G-pseudotyped SIVmac239 virus-like particles.

pCMV-ΔR8.91 and pCMV-VSV-G were used for packaging and pseudotyping of viral particles (Manel et al., 2010).

HEK293FT cells were transfected in 10 cm plates with 1.2 ml DMEM, 18 μg DNA and 48 μl TransIT-293 (Mirus Bio) per plate in 10 mL final volume to produce viral particles; for VSV-G–pseudotyped SIVmac VLPs/Vpx, 2.4 μg pTRIP CMV-VSVg and 15.6 μg pSIV3+; for shRNA viral particles, 9.6 μg pLk0.1, 6 μg pCMV-ΔR8.91, and 2.4 μg pTRIP–CMV-VSV-G; HEK293FT cells were transfected in 15 cm plates with Promega kit for calcium phosphate transfection; for X4-HIV-1-GFP, 60 μg of NL4-3 CXCR4-HIV-1-GFP; for X4–HIV-1–GAG-iGFP, 60 μg of NL4-3 CXCR4-HIV-1-iGAG-GFP. Media was changed after 1 day of transfection and viral supernatants were collected after 48 h of transfection and filtered at 0.45 μm.

### Transduction

Blood monocytes were plated at 1 × 10^6^ cells/mL in 5 mL of medium with cytokines (hGM-CSF and hIL-4) and 8 μg/ml polybrene (Merck Millipore). They were transduced with 2.5 mL of SIVmac VLP/Vpx (Mangeot et al., 2002) and 4 mL of viral supernatant containing shRNA coding lentiviruses. Fresh media and cytokines were added to cells (40% of total volume) 1 day and 3 days after CD14^+^ cell isolation. On day 4, cells differentiated into MDDCs, were collected, resuspended in fresh media with cytokines and used for transfer experiments.

### Trans-infection/HIV Transfer Experiments

After 4 days of stimulation with cytokines 50,000 MDDCs (1 million/mL) were mixed with 50,000 (1 million/mL) autologous CD4^+^ T cells and 150 μl X4-tropic HIV-1 encoding GFP (50 ng p24^GAG^, quantified by ELISA) per well in 96-well round bottom plates. Media was replaced after 24 h. Depending on experimental conditions, CD4^+^ T lymphocyte infection was measured by flow cytometry by monitoring GFP and P24 (HIV viral capsid protein) expression, following 20 and/or 40 h of co-culture. In each HIV transfer experiments, MDDC and CD4^+^ T cell viability and maturation/activation states were analyzed by flow cytometry.

### Flow Cytometry Analysis

For flow cytometry analysis, the staining was performed with anti-CD3 AF700 (for T cells) (eBioscience; 56-0038-82), anti–DC-SIGN PE (for MDDCs) (Clone, 120507; R&D systems, catalog number: FAB161P), anti-CD169 APC (for CD169/Siglec-1; Biolegend; 346007), and DAPI for live/dead gating and in some cases anti-P24 PE (Beckman Coulter, 6604667). Unless otherwise mentioned, antibodies were used at a concentration of 1/200. To assess changes in cell number, CountBright Absolute Counting Beads (Life Technologies) were used in each sample. For some experiments (transfer and/or capture), BD cytofix/cytoperm buffer and fixation protocol was used to detect intracellular P24 staining in both MDDCs and T cells. Flow cytometry was performed on a BD LSRII using a 96-well plate HTS reader. Data were then analyzed using FlowJo software (FlowJo LLC). For each donor, MDDC differentiation was monitored based on DC-SIGN induction, CD14 downregulation and minimal maturation (CD86^+^ cells <10%) [anti-CD86 PE (eBioscience, 12-0869-42)].

### Immunofluorescence and Confocal Microscopy

After different co-culture times with X4-HIV-1-Gag-iGFP and/or T cells, cells were put on coverslips previously coated with poly-L-lysine solution (0.1% w/v in H2O, Sigma), for 30 min at 37°C. Surface staining was performed in PBS containing 1% BSA, for 30 min at room temperature. Cells were incubated with warm paraformaldehyde A in PHEM Buffer [2X PHEM Buffer (500 ml): 18.14 g PIPES; 6.5 g HEPES; 3.8 g EGTA; 0.99 g MgSO4; pH to 7.0 with 10M KOH], for 10 min at room temperature. Following fixation, cells were permeabilized in Triton 0.1% diluted in PHEM buffer for 5 min at RT. Blocking was done in casein overnight at 4°C. For intracellular staining, antibodies were diluted in PBS containing 1% BSA and incubated for 1 h at room temperature. For nucleus detection, cells were then stained with DAPI (1/5,000 dilution from stock at 5 mg/ml in H2O), 5 min at room temperature. After extensive washing in PBS containing 0.1% BSA, cells were mounted in a home-made DABCO-PVA medium [2.5% Dabco, 10% polyvinylalcohol (PVA), 5% glycerol, 25 mM tris buffer pH 8,7]. Microscopy was performed on a Zeiss LSM 710 confocal with 405, 488, 543, and 633 nm lasers, a 63X N.A. 1.40 lens, and the pinhole set to 1 Airy unit as defined by Zeiss. Sequential scanning was used to assure no spillover of channels. Z series were taken at intervals of 400 nm. The following antibodies were used for confocal microscopy, at a concentration of 1/200. Primary antibodies: Mouse anti Human CD169 Alexa Fluor® 647 (AbD Serotech; MCA2517A647T); Alexa Fluor^®^ 568 Phalloidin (Molecular Probes^®^ A12380).

### Image Processing, Colocalization, and Quantification

ImageJ software was used for all image adjustments and quantification. All the pictures were smoothened and in some case CLAHE plugin (Contrast Limited Adaptive Histogram Equalization) was applied. At least 100 cells, with a minimum of 10 Z-stacks per cell, were individually analyzed for each sample. To measure and quantify dendrites length, cell shape and perimeter, there were manually drawn using the freehand line tool in ImageJ, with a width of 10. Then areas and perimeters were calculated using analyze function (measure, set measurements: Area and perimeter) for each cell on the image. Pearson correlation coefficient was calculated using ImageJ coloc2 plugging tool, in order to quantify colocalization between CD169/Siglec1 and HIV-1 in MDDCs.

### Pharmacological Drugs

All drugs were resuspended in DMSO and used at the concentrations mentioned below. CK-666 (Sigma-Aldrich, SML0006, 100 μM); Nelfinavir (Sigma-Aldrich, CDS021783, 1 μM).

Innate and inflammatory stimuli were used at the indicated concentrations: poly(I:C) (InvivoGen, 10 μg/ml); LPS (List Biological Laboratories, INC, 100 ng/ml).

### Quantitative RT–PCR

RNA was extracted from 3 × 10^5^ MDDCs using TRIZOL^®^ RNA isolation protocol for gene expression quantification. Real time quantitative PCR (qPCR) was performed after reverse transcription (RT) using a Roche LightCycler 480 with Roche 480 SYBR Green I master reagent according to manufacturer specifications. The relative abundance of each target mRNAs was calculated based on the standard curve and normalized using *GAPDH* as a control.

For qPCR, the primers used were the following:

*TSPAN7* set 1 F 5′-CCTTCGTCTTCTGGATCACTGGGG-3′;

*TSPAN7* set 1 R 5′-CATGGTCCACTGCCCGGCTC-3′;

*TSPAN7* set 2 F 5′-CATCGCTGGAGTGGCGTTTGGA-3′;

*TSPAN7* set 2 R 5′-TGCACGTTGTGGGGTAAGGGG-3′;

*GFP* F 5′- ACGTAAACGGCCACAAGTTC-3′;

*GFP* R 5′-AAGTCGTGCTGCTTCATGTG-3′.

### HIV-1 Capture Experiments

Four days after transduction with shRNAs, MDDCs were mixed with X4–HIV-1–GFP using the same conditions as with trans-infection experiments but without T cells. After 20 h co-culture, MDDCs were washed in ice cold PBS to remove unbound HIV-1 and RNA was extracted. After RT, the amount of HIV-1 RNA was detected by qPCR with primers for *GFP* (encoded inside the HIV genome) and normalized to *GAPDH*.

### RNA Sequencing

Transcriptional profiling of MDDCs was performed using whole genome RNA sequencing. MDDCs from four healthy blood donors were infected or stimulated as indicated in the figure legends, stained with anti-human CD86 (BD Biosciences) and a live/dead viability dye (ThermoFischer) before sorting into serum on a FACSAria II (BD Biosciences). Cells were lysed in TRIzol reagent (Thermo Fisher), RNA was isolated according to the manufacturer's instructions, and the samples were submitted to HudsonAlpha Institute for Biotechnology (https://hudsonalpha.org/) for library preparation and RNA-sequencing. Fifty bp-length single-end sequences were aligned to the human genome (hg38) using STAR version 2.4.2a. Samtools 0.1.19 was used to filter alignments to a MAPQ score threshold of 30. Counts per gene were called using feature Counts version 1.4.6 and gencode v24 genome annotation. Samtools 0.1.19 was used to filter alignments to a MAPQ score threshold of 30.

### Statistics

Unless otherwise specified, statistical analyses were done using the Holm-Sidak multiple comparison test following one-way Anova (NS, non-significant; ^*^p < 0.05; ^**^p < 0.01; ^***^p < 0.001; ^****^p < 0.0001). For most experiments, two different human blood donors were used and the average of triplicates and standard deviation was calculated for each blood donor.

## Data Availability Statement

The raw data supporting the conclusions of this article will be made available by the authors, without undue reservation, to any qualified researcher.

## Ethics Statement

The studies involving human participants were reviewed and approved by Etablissemnt Français du Sang (EFS), agreement N°18/EFS/030. Written informed consent for participation was not required for this study in accordance with the national legislation and the institutional requirements.

## Author Contributions

MM performed most of the experiments, wrote the manuscript, generates the figures, and obtained the funding necessary for this project. BP wrote the manuscript and performed some experiments. VG-P helped with the design of the figures and some of the experiments. ML helped writing the material and methods and provide her technical expertise with some experiments. All authors approved the manuscript for publication.

### Conflict of Interest

The authors declare that the research was conducted in the absence of any commercial or financial relationships that could be construed as a potential conflict of interest.

## Supplementary Material

Figure S1 Experimental strategy and kinetic of HIV-1 trans-infection following *TSPAN7* shRNA knockdown. Related to Figure 1. **(A)** Experimental design to evaluate the effect of viral replication on HIV-1 transfer from iMDDCs to CD4^+^ T lymphocytes. Red arrows indicate Nelfinavir (NFV, an HIV protease inhibitor) treatment at 1 μM during co-culture experiments. **(B)** Percentage of variation of X4–HIV-1–GFP transfer as compared to the 20 h time point, in the presence or absence of NFV (1 μM). Mean ± SD of technical triplicates from three different healthy blood donors. **(C–E)** Percentage of CD4^+^ T cells infected by X4–HIV-1–GFP upon co-culture with iMDDCs, transduced by scramble or *TSPAN7* shRNA (1 and 3), in the presence (black bars) or absence (white bars) of NFV (1 μM). T cell infection was measured by flow cytometry and data represent mean ± SD of technical triplicates from three healthy blood donors (donor A in **C**, donor B in **D**, and donor AC in **E**). **(E)** Data from **(D)** represented as variation of X4–HIV-1–GFP transfer as compared to MDDCs transduced by scramble shRNA, at 20 h. **(B–E)** NS, not significant. ^*^*p* < 0.05; ^**^*p* < 0.01; ^***^*p* < 0.001. **(C–E)** Donors are representatives of 8 unrelated donors tested in the context of 4 independent experiments. **(F)** Experimental design to evaluate the effect of actin nucleation inhibition on X4–HIV-1–GFP transfer from iMDDCs to CD4^+^ T lymphocytes, at 20 and 40 h, using CK-666 as an ARP2/3 inhibitor (100 μM).

Figure S2 Quantification of changes in MDDC shape and loss of dendrites. Related to Figure 2. **(A)** Arithmetic formula and illustration to assess cell shape by calculating circularity index (0 ≤ circularity ≤ 1), taking into account the area and perimeter of each cell. A value of 1 represents a cell perfectly circular, whereas 0 indicates a cell with a shape of a line. After manually tracing the perimeter of each cell, areas and perimeters were calculated using Analyze function (Analyze, measure, set measurements: area and perimeter) in ImageJ. **(B)** Box plot of circularity index calculated per cell for each group and at different time (4, 20 h and 40 h) and treatment (LPS-stimulated, *TSPAN7* shRNA-transduced or control cells). Every dot represents a cell and the number of cells analyzed in each condition is indicated on top. **(C,D)** Box plots showing the number and average length of dendrites, respectively, per cell for each group mentioned above. **(E)** Box plot displaying the length of all examined dendrites through this data analysis. Every dot now represents the measurement of one dendrite. Number of total dendrites analyzed in each condition is displayed on top.

Figure S3 *TSPAN6*and *TSPAN7* expressions relative to MDDCs maturation. Related to Figure 3. **(A)** Flow cytometry plot showing the gating strategies to analyze the level of maturation of MDDCs and percent infection of T cells. **(B)** Histogram depicting the mean fluorescent intensity (MFI) of CD86 expression in MDDCs as gated in Figure 3A (bottom panel). **(C)** Expression of *TSPAN6* and *TSPAN7* measured by bulk RNA sequencing, under different conditions: stimulation with LPS (1 μg/ml), poly(I:C) (1 μg/ml), or infection with VSV-G–HIV-1–GFP in the presence of Vpx, measured after 2, 4, 20, and 40 h post stimulation/infection. For the 20 h time point, MDDCs infected by HIV-1 were sorted on GFP^+^ and GFP^−^ populations. After 40 h, GFP^+^ MDDCs were sorted based on their level of maturation (CD86^high^ vs. CD86^low^). Data are displayed in FPKM (Fragments per Kilobase of gene per Million Reads aligned). **(D)** Schematic summarizing the inverse correlation between MDDC maturation and *TSPAN7* expression, upon co-culture experiments and the two different phases of HIV-1 transfer identified in our experimental settings.

Figure S4 Efficiency of HIV-1 transfer based on MDDC maturation status. Related to Figure 4. **(A)** Scheme depicting the experimental layout used to evaluate the effect of adding 100 ng/ml of LPS at different time before the start of co-culture with CD4^+^ T lymphocytes and X4–HIV-1–GFP (48, 24 h before or at the same time). Forty hours after the start of co-culture experiments, infected T cells were monitored by P24 and GFP expression using flow cytometry. **(B)** Histogram depicting the fluorescent intensity linked to the expression of CD86 on LPS-stimulated MDDCs vs. iMDDCs. The red line shows CD86 expression on iMDDCs and the blue one CD86 expression following LPS treatment: 48 h before co-culture (left panel), 24 h before co-culture (middle panel) and at the start of co-culture (right panel). **(C)** Quantification of HIV-1 capture by MDDCs 20 h after culture with X4-HIV-1-GFP, using two different donors. The amount of *GFP* RNA (expressed in the open reading frame of the viral protein Nef) was measured by reverse transcription and, subsequently, quantitative real time PCR with specified primers for *GFP* or *GAPDH* (housekeeping gene). Each condition is compared to the amount of *GFP* detected in immature MDDCs after 20 h. **(D)** Comparison of efficiency of HIV-1 transfer to CD4^+^ T lymphocytes when using iMDDCs vs. mMDDCs. Efficiency of transfer was calculated as follow on 2 different blood donors: ratio of captured HIV-1 by LPS-treated MDDCs compared to iMDDCs divided by the variation of transferred HIV-1 to T lymphocytes (variation of transferred HIV-1 from LPS-treated MDDCs to CD4^+^ cell compared to iMDDCs). For donor D, from Figure S4C, we measured close to 15 times more viruses captured by mature MDDCs but only 1.5 more T cells infected (Figure 4C). Transfer from mMDDCs appears to be 10 times less efficient than from iMDDCs and is therefore represented by negative values. Each bar represents mean ± SD of triplicates. **(E)** iMDDCs were treated with LPS (100 ng/mL) for 48 h before co-culture, at the time of co-culture (0 h) or left untreated. The percentages of cells with a CD169/Siglec1 and HIV-1 aggregates were evaluated in 4 unrelated donors.

Figure S5 CD169 expression in iMDDCs vs. mMDDCs. Related to Figure 5. **(A)** Cell density histogram depicting the fluorescent intensity (MFI) of CD169 expression in MDDCs detected in Figure 5A (bottom panels). The gray area represents cells stained with the isotype IgG1, the blue, red, and orange lines show CD169 expression on iMDDCs and LPS-treated MDDCs, respectively. **(B)** Diagram displaying experimental conditions and protocol to block CD169 function in HIV transfer experiments as in Figures 5B,C. **(C)** Scheme showing the experimental layout used to assess CD169 role on HIV-1 transfer at different states of MDDC maturation, as described above. **(D)** Quantification of HIV-1 capture in iMDDCs transduced with scramble shRNA and treated with an anti-CD169 antibody as compared to isotype control. The amount of X4–HIV-1-GFP captured was measured after 20 h, by quantitative real time PCR with specified primers for *GFP* and *GAPDH* (housekeeping gene). Each bar represents mean ± SD of triplicates. NS, not significant. ^**^*p* < 0.01. **(E,F)** Detailed image and magnification of confocal microscopy images, respectively, shown in Figure 5D to assess the degree of colocalization between HIV-1 (GFP) and CD169 (magenta), 4 h after the start of co-culture experiments between iMDDCs **(E)** or LPS-treated MDDCs **(F)** with X4–HIV-1–Gag-iGFP and activated CD4^+^ T lymphocytes. In **(E)**, the circles represent HIV particles detected on the dendrites or at the plasma membrane. Cellular components were stained as mentioned above. ZEISS LSM 710 microscopy. 63X/oil objective. 0.4 μM section and 0.13 μM/pixel. **(G)** Pearson coefficient was calculated for four independent donors on immature and LPS-stimulated MDDCs in order to highlight co-localization of CD169/Siglec1 and X4–HIV-1–GAGiGFP. The dotted line represents Y = 0.5. Paired *t*-test was applied (*P* < 0.05).
