# Supplementary figures and images for: Dendritic Cell Maturation Regulates TSPAN7 Function in HIV-1 Transfer to CD4+ T Lymphocytes
Source: Front Cell Infect Microbiol. 2020 Feb 28;10:70. doi: 10.3389/fcimb.2020.00070 (PMC7059179; doi:10.3389/fcimb.2020.00070)

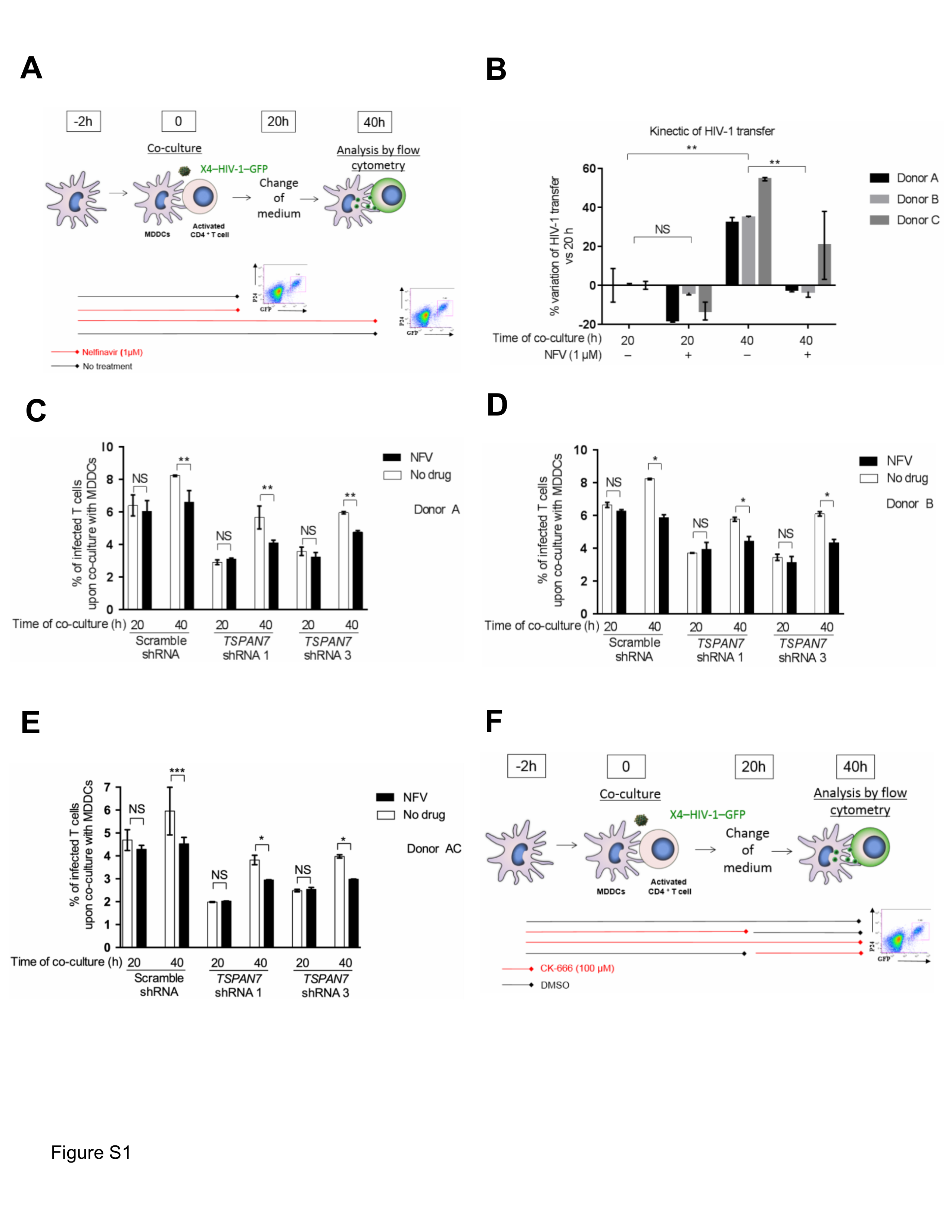

Supplement: Figure S1 — Experimental strategy and kinetic of HIV-1 trans-infection following TSPAN7 shRNA knockdown. Related to Figure 1. (A) Experimental design to evaluate the effect of viral replication on HIV-1 transfer from iMDDCs to CD4+ T lymphocytes. Red arrows indicate Nelfinavir (NFV, an HIV protease inhibitor) treatment at 1 μM during co-culture experiments. (B) Percentage of variation of X4–HIV-1–GFP transfer as compared to the 20 h time point, in the presence or absence of NFV (1 μM). Mean ± SD of technical triplicates from three different healthy blood donors. (C–E) Percentage of CD4+ T cells infected by X4–HIV-1–GFP upon co-culture with iMDDCs, transduced by scramble or TSPAN7 shRNA (1 and 3), in the presence (black bars) or absence (white bars) of NFV (1 μM). T cell infection was measured by flow cytometry and data represent mean ± SD of technical triplicates from three healthy blood donors (donor A in C, donor B in D, and donor AC in E). (E) Data from (D) represented as variation of X4–HIV-1–GFP transfer as compared to MDDCs transduced by scramble shRNA, at 20 h. (B–E) NS, not significant. *p < 0.05; **p < 0.01; ***p < 0.001. (C–E) Donors are representatives of 8 unrelated donors tested in the context of 4 independent experiments. (F) Experimental design to evaluate the effect of actin nucleation inhibition on X4–HIV-1–GFP transfer from iMDDCs to CD4+ T lymphocytes, at 20 and 40 h, using CK-666 as an ARP2/3 inhibitor (100 μM). [file Image_1.tiff]

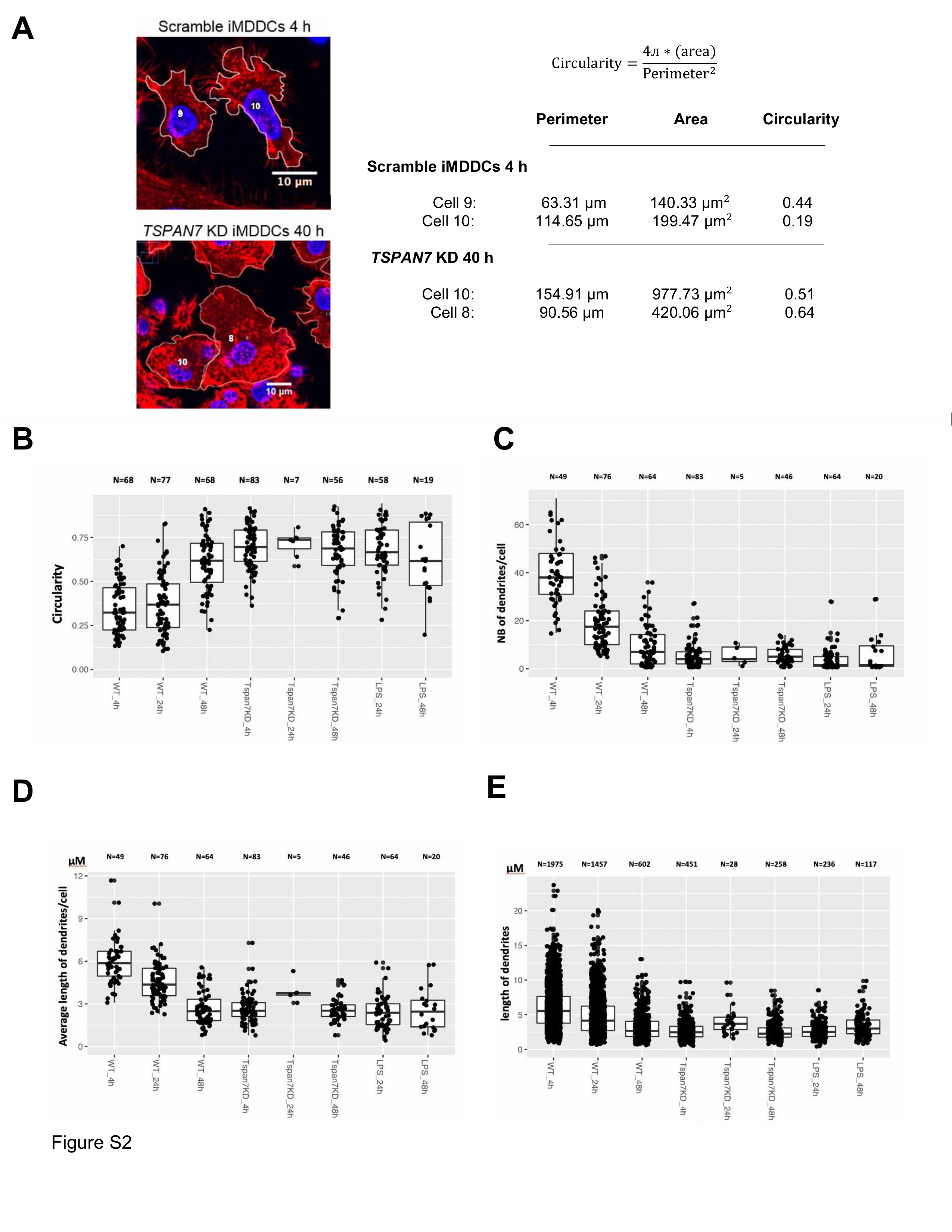

Supplement: Figure S2 — Quantification of changes in MDDC shape and loss of dendrites. Related to Figure 2. (A) Arithmetic formula and illustration to assess cell shape by calculating circularity index (0 ≤ circularity ≤ 1), taking into account the area and perimeter of each cell. A value of 1 represents a cell perfectly circular, whereas 0 indicates a cell with a shape of a line. After manually tracing the perimeter of each cell, areas and perimeters were calculated using Analyze function (Analyze, measure, set measurements: area and perimeter) in ImageJ. (B) Box plot of circularity index calculated per cell for each group and at different time (4, 20 h and 40 h) and treatment (LPS-stimulated, TSPAN7 shRNA-transduced or control cells). Every dot represents a cell and the number of cells analyzed in each condition is indicated on top. (C,D) Box plots showing the number and average length of dendrites, respectively, per cell for each group mentioned above. (E) Box plot displaying the length of all examined dendrites through this data analysis. Every dot now represents the measurement of one dendrite. Number of total dendrites analyzed in each condition is displayed on top. [file Image_2.tif]

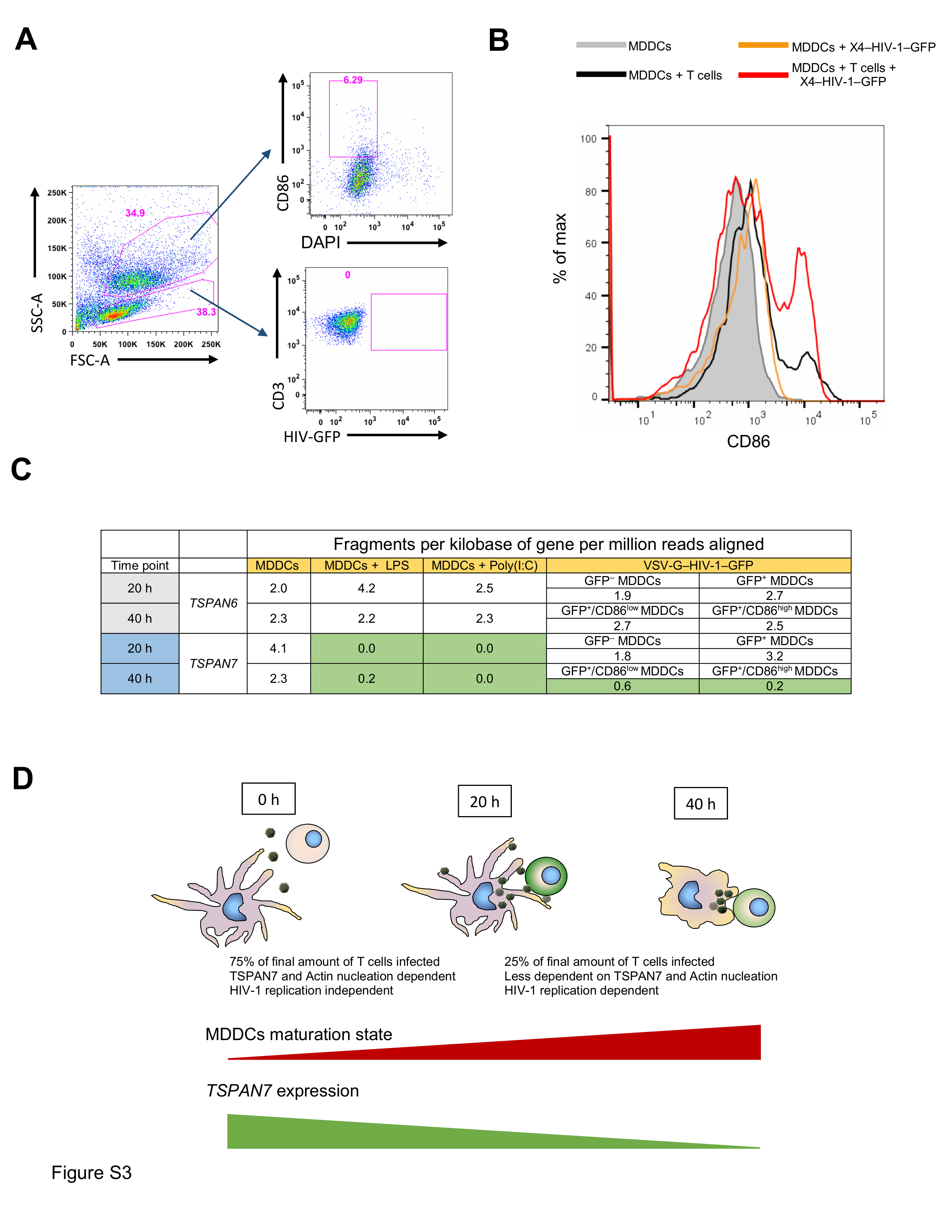

Supplement: Figure S3 — TSPAN6and TSPAN7 expressions relative to MDDCs maturation. Related to Figure 3. (A) Flow cytometry plot showing the gating strategies to analyze the level of maturation of MDDCs and percent infection of T cells. (B) Histogram depicting the mean fluorescent intensity (MFI) of CD86 expression in MDDCs as gated in Figure 3A (bottom panel). (C) Expression of TSPAN6 and TSPAN7 measured by bulk RNA sequencing, under different conditions: stimulation with LPS (1 μg/ml), poly(I:C) (1 μg/ml), or infection with VSV-G–HIV-1–GFP in the presence of Vpx, measured after 2, 4, 20, and 40 h post stimulation/infection. For the 20 h time point, MDDCs infected by HIV-1 were sorted on GFP+ and GFP− populations. After 40 h, GFP+ MDDCs were sorted based on their level of maturation (CD86high vs. CD86low). Data are displayed in FPKM (Fragments per Kilobase of gene per Million Reads aligned). (D) Schematic summarizing the inverse correlation between MDDC maturation and TSPAN7 expression, upon co-culture experiments and the two different phases of HIV-1 transfer identified in our experimental settings. [file Image_3.tif]

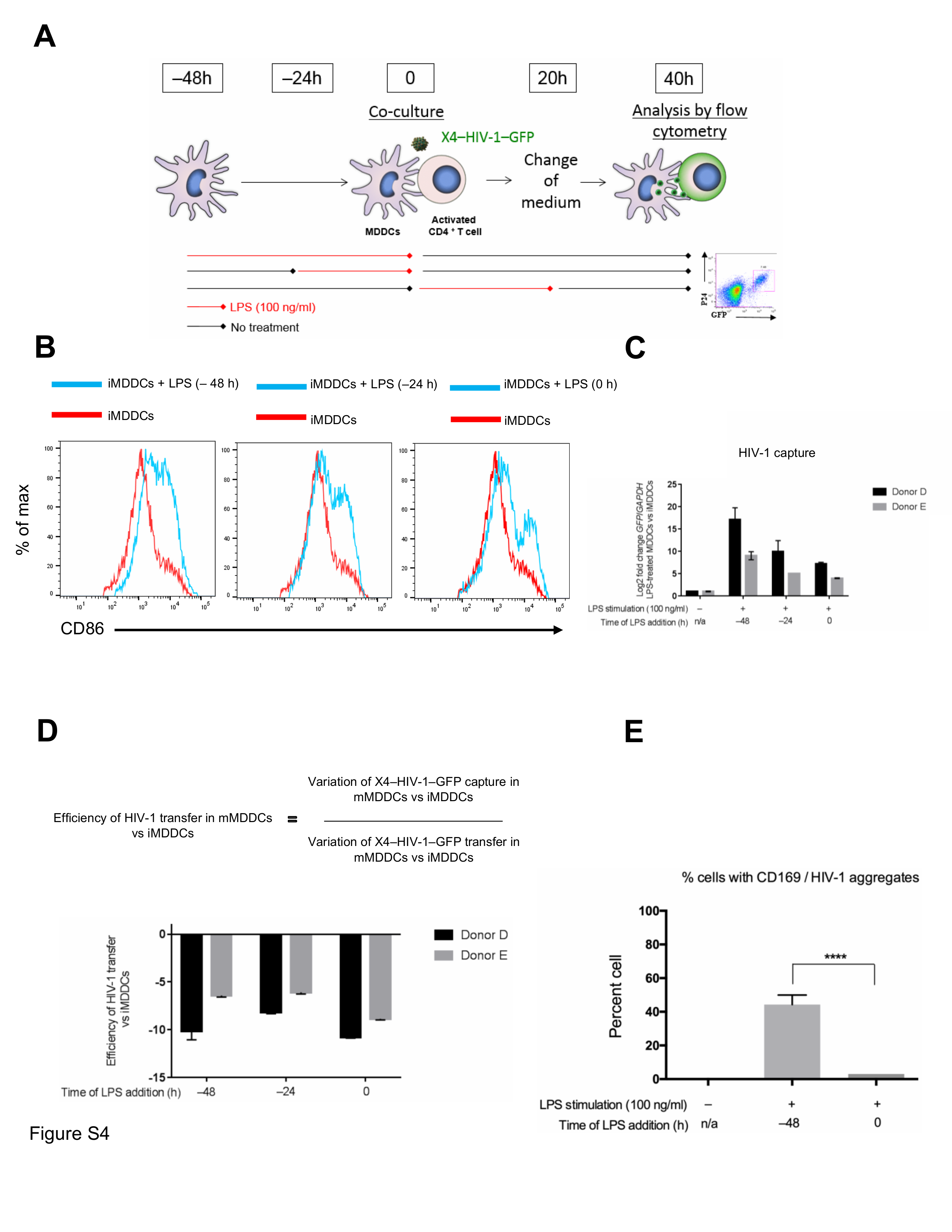

Supplement: Figure S4 — Efficiency of HIV-1 transfer based on MDDC maturation status. Related to Figure 4. (A) Scheme depicting the experimental layout used to evaluate the effect of adding 100 ng/ml of LPS at different time before the start of co-culture with CD4+ T lymphocytes and X4–HIV-1–GFP (48, 24 h before or at the same time). Forty hours after the start of co-culture experiments, infected T cells were monitored by P24 and GFP expression using flow cytometry. (B) Histogram depicting the fluorescent intensity linked to the expression of CD86 on LPS-stimulated MDDCs vs. iMDDCs. The red line shows CD86 expression on iMDDCs and the blue one CD86 expression following LPS treatment: 48 h before co-culture (left panel), 24 h before co-culture (middle panel) and at the start of co-culture (right panel). (C) Quantification of HIV-1 capture by MDDCs 20 h after culture with X4-HIV-1-GFP, using two different donors. The amount of GFP RNA (expressed in the open reading frame of the viral protein Nef) was measured by reverse transcription and, subsequently, quantitative real time PCR with specified primers for GFP or GAPDH (housekeeping gene). Each condition is compared to the amount of GFP detected in immature MDDCs after 20 h. (D) Comparison of efficiency of HIV-1 transfer to CD4+ T lymphocytes when using iMDDCs vs. mMDDCs. Efficiency of transfer was calculated as follow on 2 different blood donors: ratio of captured HIV-1 by LPS-treated MDDCs compared to iMDDCs divided by the variation of transferred HIV-1 to T lymphocytes (variation of transferred HIV-1 from LPS-treated MDDCs to CD4+ cell compared to iMDDCs). For donor D, from Figure S4C, we measured close to 15 times more viruses captured by mature MDDCs but only 1.5 more T cells infected (Figure 4C). Transfer from mMDDCs appears to be 10 times less efficient than from iMDDCs and is therefore represented by negative values. Each bar represents mean ± SD of triplicates. (E) iMDDCs were treated with LPS (100 ng/mL) for 48 h before co-culture, [file Image_4.tif]

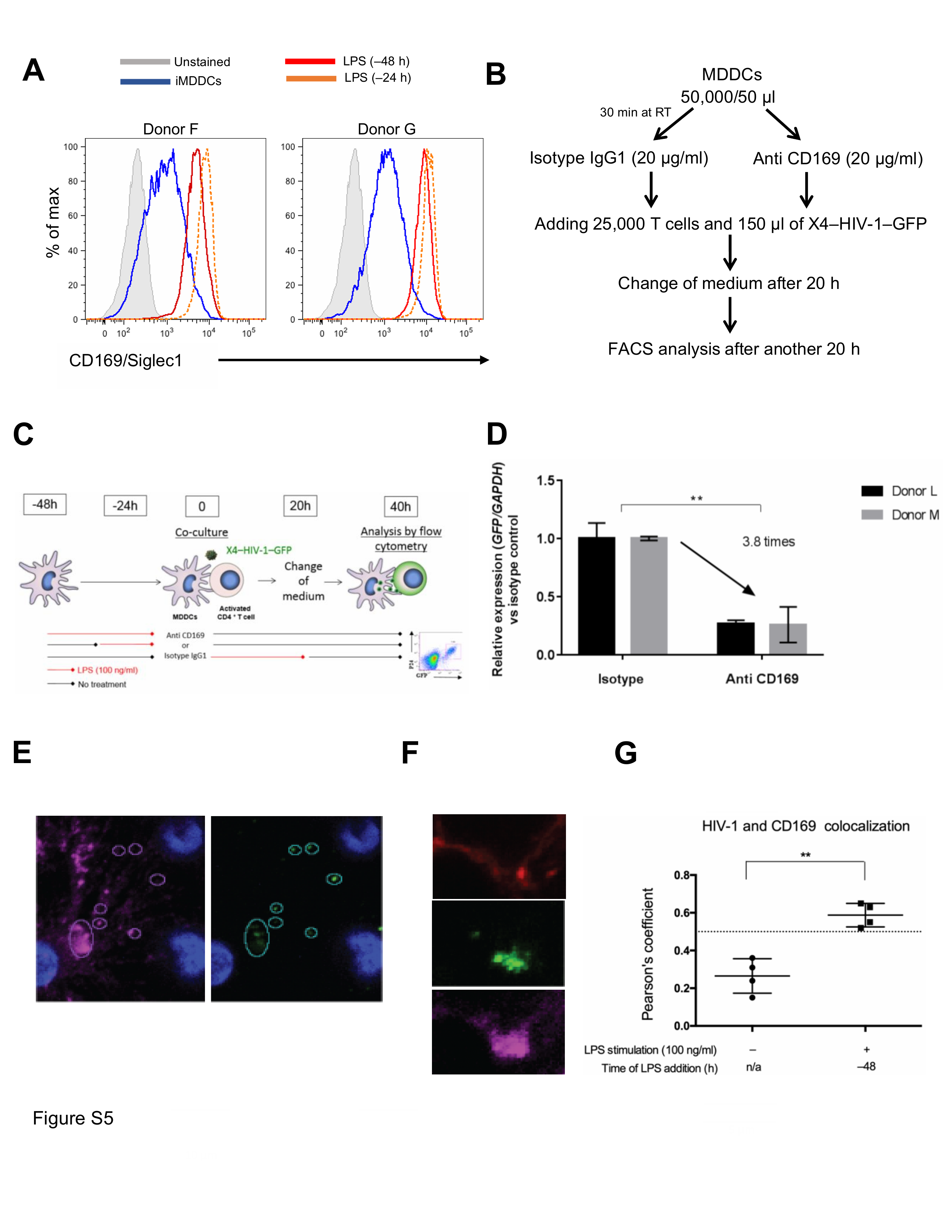

Supplement: Figure S5 — CD169 expression in iMDDCs vs. mMDDCs. Related to Figure 5. (A) Cell density histogram depicting the fluorescent intensity (MFI) of CD169 expression in MDDCs detected in Figure 5A (bottom panels). The gray area represents cells stained with the isotype IgG1, the blue, red, and orange lines show CD169 expression on iMDDCs and LPS-treated MDDCs, respectively. (B) Diagram displaying experimental conditions and protocol to block CD169 function in HIV transfer experiments as in Figures 5B,C. (C) Scheme showing the experimental layout used to assess CD169 role on HIV-1 transfer at different states of MDDC maturation, as described above. (D) Quantification of HIV-1 capture in iMDDCs transduced with scramble shRNA and treated with an anti-CD169 antibody as compared to isotype control. The amount of X4–HIV-1-GFP captured was measured after 20 h, by quantitative real time PCR with specified primers for GFP and GAPDH (housekeeping gene). Each bar represents mean ± SD of triplicates. NS, not significant. **p < 0.01. (E,F) Detailed image and magnification of confocal microscopy images, respectively, shown in Figure 5D to assess the degree of colocalization between HIV-1 (GFP) and CD169 (magenta), 4 h after the start of co-culture experiments between iMDDCs (E) or LPS-treated MDDCs (F) with X4–HIV-1–Gag-iGFP and activated CD4+ T lymphocytes. In (E), the circles represent HIV particles detected on the dendrites or at the plasma membrane. Cellular components were stained as mentioned above. ZEISS LSM 710 microscopy. 63X/oil objective. 0.4 μM section and 0.13 μM/pixel. (G) Pearson coefficient was calculated for four independent donors on immature and LPS-stimulated MDDCs in order to highlight co-localization of CD169/Siglec1 and X4–HIV-1–GAGiGFP. The dotted line represents Y = 0.5. Paired t-test was applied (P < 0.05). [file Image_5.tiff]
